# Assessment of Mothers’ Knowledge, Attitudes, and Practices Regarding Childhood Vaccination during the First Five Years of Life in Saudi Arabia

**DOI:** 10.3390/nursrep11030047

**Published:** 2021-07-05

**Authors:** Wedad M. Almutairi, Fatmah Alsharif, Fathia Khamis, Lujain A. Sallam, Loujain Sharif, Afnan Alsufyani, Fatima N. Alshulah, Rabab Alqasimi

**Affiliations:** 1 Maternity and Child Department, Faculty of Nursing, King Abdulaziz University, Jeddah 21551, Saudi Arabia; walmutairi@kau.edu.sa; 2 Medical Surgical Department, Faculty of Nursing, King Abdulaziz University, Jeddah 21551, Saudi Arabia; lsallam@kau.edu.sa; 3 Nursing Public Health, Faculty of Nursing, King Abdulaziz University, Jeddah 21589, Saudi Arabia; fkibrahim@kau.edu.sa; 4 Nursing Mental Health, King Abdulaziz University, Jeddah 21589, Saudi Arabia; lsharif@kau.edu.sa; 5 Faculty of Nursing, King Abdulaziz University, Jeddah 21589, Saudi Arabia; Aalsufyani0020@stu.kau.edu.sa (A.A.); falshulah@stu.kau.edu.sa (F.N.A.); ralqasimi0002@stu.kau.edu.sa (R.A.)

**Keywords:** vaccination, child, mothers, knowledge, attitude, Saudi Arabia, practice

## Abstract

Aim: This study’s aim was to assess the knowledge, attitudes, and practices of mothers regarding childhood vaccination during the first five years of children’s lives in Saudi Arabia. Method: A cross-sectional, descriptive study was conducted with the application of convenience sampling, and 262 questionnaires were completed by Saudi mothers who had children aged 5 years or younger. Results: The majority of the participants were aged 25–31 years (57%), 61% held a bachelor’s degree, and 60.3% had children aged 2–5 years. The knowledge score was 86%, 2492 out of a total score of 2893; the attitude score was 89.1%, 973 out of a total score of 1052; the practice score was 80.5%, 1059 out of a total score of 1315. There was no evidence of an association (*p* > 0.05) between the knowledge, attitudes, and practice of mothers regarding vaccination and their sociodemographic aspects. Conclusion: The Saudi mothers in our sample were knowledgeable, with positive attitudes regarding vaccination, and they demonstrated good practices. This might be explained by the higher educational level of our sample. Recommendations: We recommend using multiple educational methods to support the practice of mothers regarding the management of complications of vaccinations.

## 1. Introduction

Protection from diseases is one of the uttermost benefits that any country can offer to its people [1]. It is certain that vaccines are an essential part of a health system [2], an effective tool for controlling diseases in many countries around the world [3], and the most cost-effective mechanism for morbidity and mortality prevention that permits people to better protect themselves from particular bacteria and viruses [4,5,6,7,8]. In order to have the greatest protection against diseases, children should receive all their vaccinations within recommended intervals and at the appropriate age [9,10]. Vaccinating a child with appropriate vaccines would significantly reduce the costs of disease treatment and rates of disease and, therefore, improve the quality of the child’s life [11].

The level of knowledge parents have regarding child vaccination and their attitudes towards vaccination may influence their practice [12]. Major obstacles towards the high coverage of children include a lack of knowledge or information on vaccination, low levels of awareness or negative attitudes regarding vaccination, and misperceptions or rumors regarding the safety of vaccination [13,14,15].

The common factors associated with higher knowledge and attitude were the mother’s age, occupation, level of education [5], and nature of the family [5]. The most common sources of knowledge about immunization were institutions (49.5%) and internet sources (21.3%) [1,16]. Therefore, the knowledge, attitude, and practice of mothers concerning child vaccination involve a multidimensional relation that is surrounded by many variables.

In a previous study, which included 250 mothers who were selected by multistage sampling and used frequencies and percentages for statistical analysis of the data collected, 72.0% of the mothers who participated had an overall good knowledge regarding child vaccination. One hundred percent of the mothers who participated had a positive attitude regarding child vaccination. Of the mothers who participated, 98.8% stated that childhood vaccination is essential [5]. On the other hand, a study by Verulava et al. [16], which included 60 mothers and used frequencies and percentages for statistical analysis of the data collected, revealed that most of the mothers (65%) did not know the reason for the vaccinations, but they knew the right age for the vaccinations and when they must start. Fifty-nine percent believed that vaccination is not harmful. Thus, the attitudes of the mothers regarding vaccination were good, because most of the mothers believed in the importance of vaccination and they followed the vaccination schedule [2].

The results of a study by Ramadan et al. [14] indicated that 462 out of 1050 participating mothers lacked knowledge regarding obligatory vaccinations, while only six had poor attitudes toward obligatory vaccinations, and 265 of the mothers had a low practice score. Moreover, the study showed that there was a positive Pearson’s correlation (0.037) between the mother’s age and the level of knowledge. Moreover, Birhanu et al. [15] revealed that 55.0% (626) of the participating mothers had a good level of knowledge, while 53.8% had a positive attitude, and 84% a good practice regarding child vaccinations. Therefore, the study concluded that the knowledge and attitudes of the participating mothers towards child vaccination was not enough, and they recommended further health education for mothers to promote knowledge [15]. Furthermore, Sohail et al. [17], who included 200 mothers in their study, found that the mothers lacked knowledge regarding the importance of vaccination: 26.5% did not know about routine vaccinations and the vaccination schedule, and only 37.0% knew the names of the infectious diseases and when to vaccinate their children.

Mahalingam et al. [18] conducted a study including 200 mothers. Using a *t*-test, it was revealed that there was a significant difference between urban mothers and rural mothers regarding their knowledge, attitudes, and practices regarding childhood vaccinations. The study found that 75.6% of the urban mothers had a high level of knowledge compared with rural mothers. In addition, 95.9% of the urban mothers had good practice compared with rural mothers [18]. A study that included 300 mothers revealed that only 17.0% of the mothers had a good level of knowledge regarding childhood vaccinations, whereas 96.6% of the mothers had a positive attitude and 88.1% of them had good practice regarding childhood vaccination. Moreover, this study recommended more educational programs regarding childhood vaccinations [19]. Furthermore, Sunny et al. [3], who included 143 mothers in their study, found that 50.4% of mothers had excellent knowledge, whereas 34.2% had average knowledge regarding childhood vaccinations. Additionally, 64.3% of the mothers had a positive attitude, and 90.2% of the mothers had good practice towards childhood vaccinations [3].

In summary, from the studies reviewed above, it is clear that the setting where each study was conducted affected the level of knowledge, attitude, and practice regarding vaccinations at the various levels of all of the mothers and affected the correlations associated with knowledge, attitude, and practice regarding vaccinations. Moreover, most of the studies were conducted overseas.

Furthermore, it is also clear that mothers have a significant role in the care of their children and in maintaining their health status. Therefore, it is important to have a sufficient amount of knowledge, positive attitude, and correct practice regarding vaccinations. The study objectives are as follows:

1. To assess the knowledge of mothers regarding childhood vaccination during the first five years of life;
2. To assess the attitude of mothers regarding childhood vaccination during the first five years of life;
3. To assess the practice of mothers regarding childhood vaccination during the first five years of life.

## 2. Materials and Methods

### 2.1. Study Design

A quantitative, descriptive, cross-sectional approach with mothers in Saudi Arabia as subjects was used in this study.

### 2.2. Sample and Setting

Inclusion criteria were (a) mothers who have one child or more and (b) children aged from birth to five years old. Exclusion criteria included married women who did not have children, mothers who had one child or more older than five years, not living in Saudi Arabia, and not willing to participate in the study. Due to the fact of COVID-19, the research was conducted via an online survey, preventing direct contact with the participants. The online platform for the data collection was available from 1 March 2020 to 30 April 2020. The online data collection facilitated access to the target participants during the COVID-19 pandemic and strict government policy to reduce exposure. The sample size was calculated using G*power 3.1 software, which is recommended when conducting a descriptive study. The sample size was calculated using principles of power analysis, and the G*power program was used when identifying a 95% CI and the significance level of 0.05 and 50% of population. The calculation revealed that at least 200 participants were required for the data analysis in order to answer the study’s research questions. Thus, at least 200 participants were needed for this analysis.

### 2.3. Measures

The electronic questionnaire consisted of four parts. Part one included demographic characteristics (6 items), including the mother’s age, mother’s educational level, mother’s job status, mother’s monthly income, number of children, and child age group. Part two included the mother’s knowledge regarding childhood vaccination (12 items). Part three included the mother’s attitude toward childhood vaccination (4 items). Part four included the mother’s practice in relation to childhood vaccination (5 items). The participants’ responses were measured using dichotomous questions and multiple-choice questions.

Regarding the scoring of the tool, there were two possible responses, and the marks 1 and 0 were given depending on whether the response expressed having good knowledge or a positive attitude. Considering the total number of respondents, less than 50% of the total score indicated a low level of knowledge. From 50% to 75% of the total score indicated a moderate level of knowledge. Above 75–85% of the total score indicated a high level of knowledge. Regarding attitudes, lower than 50% of the total score indicated having a negative attitude toward vaccination; above 50% indicated having a positive attitude toward vaccination. Regarding practice, lower than 50% of the total score indicated having poor practice in relation to vaccination; above 50% indicated having good practice in relation to vaccination. In regard to the reliability of the tool, a pilot study was conducted with 40 mothers, and then the researchers calculated Cronbach’s alpha coefficient for the tool; it was 0.89. This indicated that the tool was highly reliable. Regarding the validity of the tool, the instrument used in the current study was designed following the method of another study [18]; the researchers received approval from the author to use the tool via email.

### 2.4. Ethical Considerations

Ethical approval was obtained from the ethical committee of the Faculty of Nursing, King Abdulaziz University Hospital in Jeddah, Kingdom of Saudi Arabia. Participation in this current study was voluntary, and names were not recorded anywhere on the questionnaire. The researcher explained the aim of the study through the statement on the online survey. Participants first signed an online consent for approval before answering the survey, and they could withdraw anytime while answering the questions. Ethics approval was granted by the college’s ethics board (Reference No. 2B.30).

### 2.5. Data Analysis

The results were computed and analyzed using the Statistical Package for the Social Sciences (SPSS) 26.0 version (IBM Inc., Chicago, IL, USA). To describe the variables, descriptive statistics were used, including means and standard deviations. For inferential statistics, Pearson’s correlation coefficient was performed to test the strength of relationships between continuous variables. Cronbach’s alpha was used for the overall knowledge and anxiety score. A Cronbach’s alpha coefficient greater than 0.70 was considered acceptable.

## 3. Results

### 3.1. Participants

A total of 262 participants (mothers) were included in this study. The majority of the participants (149; 56.9%) were 31 years or older. Approximately 61.8% (162 mothers) had a bachelor’s degree or a higher academic qualification; 56.5% (148) of the mothers were housewives. The majority of the participants deemed their monthly income satisfactory (122; 46.6%). Most of the mothers (90; 34.4%) had 2–3 children, 33.2% had more than three children, and 32.4% had only one child. The age of most of the children ranged from 2 to 5 years old (60.3%), as indicated in Table 1.

### 3.2. Assessment of the Mothers’ Knowledge Regarding Childhood Vaccination

The mothers participating in the study showed a high level of knowledge regarding vaccinations, as shown in Table 2. A total of 98.5% of the mothers ensured that their children received the obligatory vaccines. Moreover, the majority of mothers (98.5%) deemed vaccination to be very important for children from birth. In addition, 90.1% of them stated that vaccinations prevent infectious disease; decrease the rate of mortality and disabilities (93.9%) and maintain child health (97.3%); control diphtheria, tetanus, pertussis (85.9%), and measles (96.6%); prevent hepatitis B infection (96.2%). Approximately 85.9% of the mothers knew that some vaccines are associated with pain and fever, while 59.2% declared that vaccination is not supposed to cause convulsions or skin rashes. The majority of mothers (98.5%) assumed that even healthy children require vaccination. Approximately 60.1% of mothers stated that they obtained their information about vaccines from the primary healthcare center (Table 2).

### 3.3. Assessment of the Mothers’ Attitudes towards Childhood Vaccination

The participating mothers in the study showed positive attitudes towards childhood vaccination, as the score of the participants in the subscale of attitude was 973 (above 80%) of the total score (1052), which means that the participating mothers had a positive attitude towards childhood vaccination. The researchers computed the frequencies and percentages for the related items. In total, 95.0% of the participating mothers think that vaccinations are beneficial; 83.2% of the participating mothers feel that it is safe to have their child vaccinated; 93.5% of the participating mothers support the compulsory vaccination programs designed by the Ministry of Health; 98.1% of the participating mothers advise their relatives and family to vaccinate their children. The assessment of mothers’ attitude towards childhood vaccination is shown in Table 3.

### 3.4. Assessment of the Mothers’ Practice towards Childhood Vaccination

As demonstrated in Table 4, the participating mothers in the study showed good practice in relation to childhood vaccination, as the score of the participants in the subscale of practice was 1059 (from 50% to 75%) of the total score (1315), which means that the participating mothers conducted good practice in relation to childhood vaccination. The researchers computed the frequencies and percentages for the items measuring vaccination practice.

In total, 98.5% of the participating mothers had their children vaccinated with the mandatory childhood vaccines; 93.5% of the participating mothers followed the compulsory vaccination programs listed in the vaccination schedule; 41.6% of the participating mothers looked for other vaccines available to their child; 82.8% of the participating mothers applied a cold compress to reduce swelling after vaccination; 88.3% of the participating mothers used pain relievers to relieve swelling and pain after having their child vaccinated.

### 3.5. Association among Knowledge, Attitude, and Practice and Demographics of the Mothers

The following table (Table 5) indicates that there was no association between the participating mothers’ knowledge, attitudes, and practice regarding vaccination and their sociodemographic aspects (*p* > 0.05). A positive Pearson’s correlation was conducted.

Table 5 indicates that the *p*-value of the test was more than 0.05; thus, we accepted the null hypothesis and rejected the alternative one, denoting that there was not a significant difference between KAP and the demographics of the participating mothers with a level of confidence of 95%.

## 4. Discussion

The current study revealed that participants had a high level of knowledge regarding child vaccinations. This may be due to the recent efforts and procedures exerted by the Ministry of Health in Saudi Arabia over the past years. A very wide range of research refuted the claims of having lower vaccination rates in Saudi Arabia. For example, Alshammari et al. [20], who conducted a study in Saudi Arabia in 2018, concluded that most Saudi parents had good KAP towards immunization that was not associated with gender and educational degrees. In addition, Alfahl et al. [21], who conducted a study in Saudi Arabia, concluded that the majority of parents had good practice towards vaccination, with 92.8% reporting vaccination of their children according to the vaccination schedule. Moreover, a report by the WHO and UNICEF, published on 6 July 2020 on Saudi national immunization coverage, revealed that coverage of bcg was 98%, 96% for dpt1, 96% for dpt3, 97% for pol3, 96% foripv1, 95% formcv1, 96% for mcv2, 95% for rcv1, 96% for hepbb, 97% for hepb3, 96% for hib3, 95% for rotac, and 96% for pcv3 [22]. The results of the current study revealed the same results by showing very similar percentages (98.5%), as the participating mothers in the current study gave their children the mandatory children’s vaccines. Therefore, the results of the current study align with previous studies that were recently conducted in Saudi Arabia and with internationally trusted reports. The same aspects of a high level of knowledge, attitude, and practice were presented in other worldwide studies [4,5].

The current study varied from some of the existent body of research. In one study [16] that was conducted in Georgia, USA, in 2020, only 58% of the participants agreed that diseases can be prevented through vaccination, while the current study revealed that 90.1% of the participating mothers agreed that vaccination prevents infectious diseases. This means that parents in Saudi Arabia have better knowledge than that of the American parents sampled in that particular study. Contrary to the results of the current study, another overseas study [2] revealed that only 58.33% of mothers believed that vaccines are not harmful. Furthermore, in a study [14] conducted in Egypt, only 78.7% of participants agreed that vaccination reduces the child mortality rate, while the current study revealed that 93.9% of the participating mothers agreed that vaccination reduces death and disability. One of the overseas studies [2] revealed that 91.66% of participants knew that it is important to follow vaccination schedule. On the other hand, the current study revealed that 95.0% of the participants thought that vaccinations are beneficial. This indicates more positive attitudes toward vaccination.

Contrary to a Saudi Arabian study [12] that revealed that parents’ attitudes towards vaccination were positive (34.2%), except in some aspects related to vaccination’s side effects, and the chance of the occurrence of diseases against which the child was vaccinated was 39.4%, in the current study, the score of the participants on the sub-scale of attitude towards child vaccination was 973 (above 85%) out of the total score (1052), which means that the respondents had positive attitudes toward childhood vaccination for all the domains of attitude towards vaccination.

In a previous Saudi study [21], which revealed that 73.6% of the participants followed the compulsory vaccination programs listed in the vaccination schedule and that 59% of the participants used pain relievers to relieve swelling and pain after vaccinating her child, considering the date of this study (i.e., 2017), we noticed an improvement in the practice of child vaccination. The current study revealed that participating mothers had good practice towards vaccinations (93.5%) by following the compulsory vaccination programs listed in the vaccination schedule and that 88.3% of the participants used pain relievers to relieve swelling and pain after vaccinating their children.

A study [12] conducted in Saudi Arabia in 2018 revealed that parents had good knowledge on aspects related to the general role of vaccination in the prevention of some infectious diseases (91.9%), and in good agreement, the current study revealed that 90.1% of the mothers agreed that vaccination prevents infectious diseases. The same study revealed that participants agreed it is recommended to vaccinate children against some diseases such as seasonal influenza (45.7%). In comparison, in the current study, 85.9% of the mothers agreed that diphtheria, tetanus, and pertussis are controlled through vaccinations. Moreover, 96.2% of the mothers agreed that the hepatitis B virus is prevented by vaccination. Furthermore, 96.6% of the mothers stated that childhood vaccinations control measles.

In alignment with another study [21] that was conducted in Saudi Arabia in 2017 and which revealed that most of the participants (58.1%) received their information regarding child vaccination from medical staff, followed by social media (17.4%), and books (14%), the current study revealed that 60.1% of the mothers received information regarding vaccinations from primary health care centers. The same study found that only 40% of participants agreed that routine immunization protects children against infectious diseases and their complications. In comparison to this result, the current study revealed that 90.1% of the participants agreed that vaccination prevents infectious diseases, and 77.9% of the participants of the same study believed that vaccination is not generally harmful for children, while in the current study, 97.3% of the mothers agreed that vaccination keeps a child healthy. In another study [15], it was revealed that only 48.2% of mothers obtained their information regarding vaccinations from health professionals, while in the current study, 60.1% of mothers obtained their information from primary health care centers.

The study [16] that was conducted in Georgia, USA, in 2020, revealed that the majority of the interviewed mothers (97%) showed a positive attitude towards immunization. The current study revealed more positive attitudes towards childhood vaccination. For example, 98.1% of the mothers in the current study stated that they advised their relatives and family to vaccinate their children. In an international study [5], the results indicated that all of the participating mothers (100%) had a positive attitude toward immunization. This aligns with the results revealed in the current study, where 95.0% of the mothers thought that vaccinations are beneficial. Moreover, 93.5% of the sample study supported the compulsory vaccination programs.

Another Saudi Arabian study [21] revealed that 86.4% of the participants thought that vaccinations are beneficial, while in the current study, 95.0% of the mothers thought that vaccinations are beneficial. The study also revealed that 83.3% of participants felt safe when they had their children vaccinated. This is almost identical to the results from the current study, where it was revealed that 83.2% of the mothers felt safe when they had their children vaccinated. Of the former study’s participants, 92.9% supported the compulsory vaccination programs designed by the Ministry of Health, while in the current study, it was revealed that 93.5% of the mothers supported the compulsory vaccination programs designed by the Ministry of Health. The two results are almost identical. In addition, a study [19] that was conducted in Lagos State shows that 94.2% of mothers recommended vaccination to their relatives, while in the current study, 98.1% advised their relatives and family to vaccinate their children. In an overseas study [5], the results indicated that the majority (86.4%) of the respondents had fully immunized their children. This aligns with the results revealed in the current study, where 93.5% of the mothers followed the compulsory vaccination programs listed in the vaccination schedule. On other hand, one study [17] stated that only 76% of the children were fully immunized.

From our point of view, the abovementioned differences in knowledge among mothers in different geographical areas are due to the fact of a low level of education regarding vaccinations given in hospitals after birth, as today, all births take a place in hospitals or primary healthcare centers, where mothers follow the vaccination schedule. Furthermore, we also believe the information gained by mothers in hospitals or primary healthcare centers could affect their attitudes and practice regarding vaccinations. Furthermore, due to the misperceptions or rumors regarding the safety of vaccinations that arise from media and the Internet, social media may have a considerable (negative or positive) impact on the knowledge of mothers. Moreover, the large number of internet pages and websites could affect the accuracy of information regarding vaccination, which, consequently, could also affect the practice and attitude of mothers.

## 5. Limitations

As researchers, the COVID-19 pandemic and lockdown affected our ability to reach samples. This was the main motivation behind choosing the convenience sampling technique. Among the limitations of this sampling technique was its inability to represent the whole population. Another limitation of the current study lies in the geographic area. The researchers suggest further studies that make comparisons between different areas such as urban and rural areas in Saudi Arabia. This variable was not considered in the current study, despite its high importance. Moreover, this study might have some bias, as the present study was an online survey and might be limited or approached more easily by those with a higher level of education.

## 6. Study Implications

The current research recommends more effort from authorities to reach an advanced level of knowledge. These efforts can be made via educational programs on social media, as it is an effective channel for developing knowledge about vaccines. Moreover, the study recommends developing a notebook that includes advanced information about vaccinations. As the majority of births currently take place at hospitals, every mother should be provided a brief notebook about vaccinations at these hospitals. A brief educational session conducted by primary healthcare centers for mothers who are scheduled to vaccinate their children could also be arranged.

## 7. Conclusions

The majority of mothers in our Saudi Arabian sample had a high level of knowledge regarding childhood vaccinations, except in relation to two aspects: the first was that only 45.4% of the mothers agreed that malnutrition, low fever, and diarrhea are not a contraindication to vaccination; the second aspect was that only 40.8% of mothers agreed that vaccinations cause cramps and rashes. Furthermore, most mothers in Saudi Arabia had a positive attitude and conducted good practice regarding childhood vaccination. Moreover, the overall knowledge, attitudes, and practice were good among most of the mothers at 98.9%. The current study indicates that there was no association between the participating mothers’ knowledge, attitudes, and practice regarding vaccination and their sociodemographic aspects.

## Figures and Tables

**Table 1:** Distributions according to the sociodemographic characteristics of the study’s participants.

Sociodemographic Characteristics of the Mothers		Count *n*	Percent %
1. Mother’s age	From 18 to 25	56	21.4%
	From 25 to less than 31	57	21.8%
	From 31to less than 35	50	19.1%
	35 or older	99	37.8%
2. Mother’s educational level	Literate	5	1.9%
	Primary school	3	1.1%
	Secondary school	4	1.5%
	High school	88	33.6%
	Bachelor or higher	162	61.8%
3. Job status	Student	42	16.0%
	Employee	72	27.5%
	Housewife	148	56.5%
4. Monthly income	Enough	122	46.6%
	Partly enough	115	43.9%
	Not enough	25	9.5%
5. Number of children	One	85	32.4%
	Two to three	90	34.4%
	More than three	87	33.2%
6. Age of children	From 0 to 6 months	31	11.8%
	From 6 to 12 months	36	13.7%
	From 12 to 24 months	37	14.1%
	From 2 to 5 years	158	60.3%

*N* = 262. This table uses descriptive statistics: frequency (*n*) and percentage (%).

**Table 2:** Assessment of the mothers’ knowledge of childhood vaccination.

Variable	Count	Layer Total *N*%	%
Has your child received the mandatory vaccines?	Yes	258	98.5%
	No	4	1.5%
Is vaccination important for children from the first day of birth?	Yes	258	98.5%
	No	4	1.5%
Does vaccination prevent infectious diseases?	Yes	236	90.1%
	No	26	9.9%
Does vaccination reduce death and disability?	Yes	246	93.9%
	No	16	6.1%
Can vaccination keep children healthy?	Yes	255	97.3%
	No	7	2.7%
Can diphtheria, tetanus, and pertussis be controlled through vaccinations?	Yes	225	85.9%
	No	37	14.1%
Can hepatitis B virus be prevented by vaccination?	Yes	252	96.2%
	No	10	3.8%
Can childhood vaccinations control measles?	Yes	253	96.6%
	No	9	3.4%
Are malnutrition, low fever, and diarrhea not contraindications to vaccination?	Yes	119	45.4%
	No	143	54.6%
Are some vaccinations related to fever and pain?	Yes	225	85.9%
	No	37	14.1%
Can vaccinations cause cramps and rashes?	Yes	107	40.8%
	No	155	59.2%
Does even a healthy child need vaccinations?	Yes	258	98.5%
	No	4	1.5%
What is the source of your information about vaccinations?	Primary healthcare center	158	60.1%
	Friends and relatives	49	17.9%
	Ministry of health website	40	14.8%
	Study	10	3.3%
	Search	5	3.9%

*N* = 262. This table uses descriptive statistics: frequency (*n*) and percentage (%).

**Table 3:** Assessment of the mothers’ attitudes towards childhood vaccination.

Variable	Count	Layer Total *N*%	Variable
Do you think vaccinations are beneficial?	Yes	249	95.0%
	No	13	5%
Do you feel that it is safe to have your child vaccinated?	Yes	218	83.2%
	No	44	16.8%
Do you support the compulsory vaccination programs designed by the Ministry of Health?	Yes	245	93.5%
	No	17	6.5%
Do you advise your relatives and family to vaccinate their children?	Yes	257	98.1%
	No	5	1.9%

*N* = 262. This table uses descriptive statistics: frequency (*n*) and percentage (%).

**Table 4:** Assessment of the mothers’ practice in relation to childhood vaccination (*N* = 262).

Variable	Count	Layer Total *N*%	Variable
Has your child received the mandatory childhood vaccines?	Yes	258	98.5%
	No	4	1.5%
Do you follow the compulsory vaccination programs listed in the vaccination schedule?	Yes	245	93.5%
	No	17	6.5%
Do you look for other vaccines available to your child?	Yes	217	82.8%
	No	45	17.2%
Do you use pain relievers to relieve swelling and pain after having your child vaccinated?	Yes	231	88.3%
	No	31	11.7%

**Table 5:** Association between KAP and the demographics of the participating mothers (*N* = 262).

Sociodemographic Characteristics		KAP		*p*-Value
		Good (*n* = 259)	Poor (*n* = 3)	
1. Mother’s age	From 18 to 25	56	0	0.172
	From 25 to less than 31	57	0	
	From 31 to less than 35	50	0	
	35 or older	96	3	
2. Mother’s educational level	Literate	5	0	0.997
	Primary school	3	0	
	Secondary school	4	0	
	High school	87	1	
	Bachelor or higher	160	2	
3. Job status	Student	42	0	0.291
	Employee	70	2	
	Housewife	147	1	
4. Monthly income	Enough	120	2	0.730
	Partly enough	114	1	
	Not enough	25	0	
5. Number of children	One	84	1	0.999
	Two to three	89	1	
	More than three	86	1	
6. Age of children	From 0 to 6 months	31	0	0.500
	From 6 to 12 months	35	1	
	From 12 to 24 months	36	1	
	From 2 to 5 years	157	1	

## Data Availability

The data are available from the authors on request.
